# An Adaptive Deep Learning Framework for Dynamic Image Classification in the Internet of Things Environment

**DOI:** 10.3390/s20205811

**Published:** 2020-10-14

**Authors:** Syed Muslim Jameel, Manzoor Ahmed Hashmani, Mobashar Rehman, Arif Budiman

**Affiliations:** 1 Department of Computer and Information Sciences, Universiti Teknologi PETRONAS (UTP), Seri Iskandar 32610, Malaysia; manzoor.hashmani@utp.edu.my; 2 Centre for Research in Data Science (CERDAS), UTP, Perak 32610, Malaysia; 3 High Performance Cloud Computing Centre (HPC3), UTP, Seri Iskandar 32610, Malaysia; 4 Institute of Electrical and Electronics Engineers (IEEE), New Jersey, NY 10016, USA; 5 Faculty of Information and Communication Technology, Universiti Tunku Abdul Rahman, Kampar 31900, Malaysia; mobashar@utar.edu.my; 6 Faculty of Computer Science, University of Indonesia, West Java Depok 16424, Indonesia; arif.budiman21@alumni.ui.ac.id

**Keywords:** adaptive deep learning algorithm, dynamic image classification, Internet of Things (IoT), concept drift, high dimensional stream analysis

## Abstract

In the modern era of digitization, the analysis in the Internet of Things (IoT) environment demands a brisk amalgamation of domains such as high-dimension (images) data sensing technologies, robust internet connection (4 G or 5 G) and dynamic (adaptive) deep learning approaches. This is required for a broad range of indispensable intelligent applications, like intelligent healthcare systems. Dynamic image classification is one of the major areas of concern for researchers, which may take place during analysis under the IoT environment. Dynamic image classification is associated with several temporal data perturbations (such as novel class arrival and class evolution issue) which cause a massive classification deterioration in the deployed classification models and make them in-effective. Therefore, this study addresses such temporal inconsistencies (novel class arrival and class evolution issue) and proposes an adapted deep learning framework (ameliorated adaptive convolutional neural network (CNN) ensemble framework), which handles novel class arrival and class evaluation issue during dynamic image classification. The proposed framework is an improved version of previous adaptive CNN ensemble with an additional online training (OT) and online classifier update (OCU) modules. An OT module is a clustering-based approach which uses the Euclidean distance and silhouette method to determine the potential new classes, whereas, the OCU updates the weights of the existing instances of the ensemble with newly arrived samples. The proposed framework showed the desirable classification improvement under non-stationary scenarios for the benchmark (CIFAR10) and real (ISIC 2019: Skin disease) data streams. Also, the proposed framework outperformed against state-of-art shallow learning and deep learning models. The results have shown the effectiveness and proven the diversity of the proposed framework to adapt the new concept changes during dynamic image classification. In future work, the authors of this study aim to develop an IoT-enabled adaptive intelligent dermoscopy device (for dermatologists). Therefore, further improvements in classification accuracy (for real dataset) is the future concern of this study.

## 1. Introduction

The potential opportunities offered by the abundance of sensors, actuators and communications in the Internet of Things (IoT) environment produces massive and non-stationary input (image) data [1], which demands real-time, online and adaptive analysis approaches [2]. More specifically, real-time and dynamic image classification has manifested as an imperative requirement for analyzing the several critical applications [3]. However, the static nature of existing deep learning algorithms (for image classification) are not appropriate for such a dynamic environment and requires adaptive approaches to handle the changes (concept drift) during dynamic image classification tasks [4]. Such issues are particularly prominent in applications deployed over a non-stationary stream where data is continuously provided to the system. Many studies [5,6,7,8,9] in the past decade have focused on concept drift challenges when performing classification over a data stream. However, most of the studies proposed solutions are for non-imaging data streams (which possess low dimensional data). In previous studies [10,11], the authors of this study have highlighted the issue of concept drift for high-dimensional (imagery) streams. More specifically, in a study [11] the authors have presented few potential types of concept drift (novel class arrival and class evolution) issues, considering a stream of images whose labels are predicted on-the-fly for automatic categorization. Similar problems have also been addressed in the literature as a critical issue during online learning [12,13,14,15]. A recent study [12] discussed the automatic organization of images occurring on social media websites or search engines as a typical application. This study explains that traditional image classifier (trained on a known set of classes) substantially decreases the performance and becomes obsolete over time. The possible reason for this performance degradation is due to the change in image patterns or unknown image samples. In the scenarios mentioned above, the streaming image input may differ from those used to train the classifier, whereas this study only proposes the change detection mechanism for such changes.

Furthermore, some studies [14,15], also discussed the possible issue of concept drift during imagery stream analysis, such as real-time social media application (SMA). SMA, by classifying the registered user images, could recognize user faces in the future. The future images of the user may change due to aging, a haircut or some other alteration that has occurred in the face of users (virtual drift). The sample features may be as points, edges or objects. Therefore, in the case of SMA, the features of the existing users may vary, or the new user class can be introduced (real drift), or both conditions can take place at same step (hybrid drift). Because at the required training time, machine learning models are trained through extracted features from the training dataset in the form of feature vectors. A learner can also only classify the testing dataset (image), based on existing provided features-based knowledge. However, in online SMA, the features offered at the time of training might change at different timesteps, which will adversely affect the classification performance of the existing models or may make them obsolete (not capable of handling new changes). In the existing studies, streaming images differ from those used to train the classifier and mostly address the two challenges typically relying on mechanisms, namely (1) detecting changes in a data stream and (2) adopting the underlying modifications accordingly. However, it is essential to figure out the possible deterioration of the model caused by high dimensional data stream, such as hyperspectral, multispectral and colour imagery streams. In a study [13], authors have addressed such issue for complex multispectral image analysis, where authors proposed a framework (adaptive CNN ensemble) to adapt the new spectral bands arrival during multispectral image classification. However, the proposed framework was limited only for the spectral band adaptation.

In summary, the current solutions for handling uncertainty in streaming data have mostly focused on one aspect, like highlighting the importance of concept drift handling or providing a change detection mechanism or proposing an adaptive approach or focusing on the specific type of concept drift (not generic). Interestingly, most of the studies don’t provide a mechanism to perform on-the-fly learning (learning while the model is already deployed) and perform offline learning (offload model and retrain) which is not desirable. Hence, it is essential to provide a complete framework (a perfect solution including detection, adaptation and online learning/on-the-fly learning), which is able to detect changes and adapt the changes after model deployment (on-the-fly) for a variety of concept drifts in high-dimensional streams. Therefore, in this study, the authors present an improved version of a previous framework called as ameliorated adaptive CNN ensemble framework. The improvements in the current proposed framework make it generic enough to also tackle the novel class arrival and class evolution issue during online image classification.

### Contributions

The following are the key contributions of this study:

(1) To propose an ameliorated (improved) version of the adapted CNN ensemble framework to handle novel class and class evolution issue during online imagery stream.
(2) To validate the classification performance (in terms of accuracy) of ameliorated framework after novel class and class evolution using the benchmark and real dataset.
(3) To evaluate the performance of proposed ameliorated framework with state-of-the-art shallow learning (ensemble SVM and random forest) and deep learning (ensemble CNN) models.

Section 2 of this study briefly surveys the related work and formulates theoretical foundation of novel class arrival and class evolution issues. Section 3 proposes an ameliorated adaptive CNN ensemble framework to handle the novel class and class evolution problem. Section 4 discusses the experiments and obtained results in detail. More specifically, in Section 4, the proposed model was evaluated and a comparative analysis with state-of-the-art image classification models is presented. Finally, Section 5 presents the conclusions and future work.

## 2. Related Work and Theoretical Foundation

In the literature several studies have proposed unsupervised learning-based approaches to detect the novel class arrival. An earlier study [16] suggested a cluster-based novel class detection technique for stream data classification. This study only concentrated on the single class problem (it is assumed that only one class is known, and the rest of the classes are novel). Furthermore, the provided solution was only applicable to binary classification tasks. Later, numerous attempts have been made to improve this approach further. For example, a study [17] proposed the novel class detection technique if the total number of classes in streams are variable (not fixed) and has proven empirically the effectiveness of this solution for multi-class classification. This study mainly used the K-means clustering approach for novel class detection. This study proposed a q-NSC technique, which is outlier detection from the dense clusters and density measure (intra-class and inter-class) in observed feature space. A similar idea was adopted in the SAND framework [18] for novel class detection. SAND detects the novel class arrival by analyzing the confidence in predicting instances from evolving data streams. The SAND approach facilitates identifying the novel class and class evolution issues by monitoring the class performance. Results showed that SAND is an effective solution despite the fact it only uses a limited amount of labelled data. However, SAND is computationally expensive due to exhaustive invocation of the change detection module. Later, to overcome the SAND deficiency, an efficient framework was proposed by [19]. This framework was based on SAND approach, which exploits the dynamic programming, and executes the change detection module selectively to reduce the computational complexity.

Another existing study [20] presented the matrix sketches-based approach (SENCMaS) to overcome the novel class detection issue. SENCMaS measures the distance that is far away from all various directions in the global sketch. The core contribution of this study was to: (1) detect the emerging new classes, (2) classify the trained classes and (3) update the model in the streams by maintaining the low-dimensional matrix sketches (it continuously updates after further improvement). However, the existing approaches have a strong assumption about intra-class cohesion and inter-class separation property in the data and these approaches fail to hold the same approach in a high dimensionality scenario. Besides, these approaches also do not address the feature-wise changes (class evolution) in sample data feature space.

Recently, several studies have applied neural network-based methods to handle the novel class adaptation issue. More specifically, the state-of-art convolutional neural network (CNN)-based approaches have been found very useful to handle high dimensionality datasets due to CNNs’ capability to extract low (pixel) level features. A study [21] demonstrated a softmax prediction probability baseline for error and outline detection to identify the newly arrived classes in data streams. The proposed abnormality module provided a more reliable way to discriminate the existing classes and new classes by performance degradation of the classifier. This study showed high confidence probabilities (+90%) on Gaussian noise samples from the MNIST dataset. Besides, a few studies [22,23] proposed an enhanced version of this approach using the temperature scaling mechanism and input preprocessing to segregate the existing and newly arrived classes. The input data preprocessing mechanism is achieved using slight perturbations on the direction of the input instances’ gradients. These studies focused on the high dimensional datasets such as imagery datasets (MNIST and CIFAR-10), but yet does not cover the adaptability or online training mechanism adequately. In 2018, a study [24] proposed a semi-supervised stream classification framework that utilizes a CNN classifier called convolutional open-world classifier. This framework was an adaptive solution to handle the novel class arrival issue and mainly covered the imagery streams (greyscale, and RGB images) with an intrinsic high-quality similarity metric, which is trained using multitask learning. However, this solution only focused on novel class adaptation (not on class evolution or new sample arrival). Moreover, it did not cover the online training aspect (which is the primary factor for adaptation). Mostly, in literature, the online training is achieved for low dimensionality data using some incremental learning strategy. Typically, incremental learning is applied in a scenario where new classes are evolved continuously. In the case of novel class arrival, only a few classes are available in the beginning, and new classes emerge later.

Based on an intensive literature review on the relevant topic, this study concludes that the issue of novel class and class evaluation has been highlighted as a critical problem during high-dimensionality stream analysis. In existing studies, the provided solutions are mainly focused on the novel class detection problem (to detect arrival of a new class) and ignore the adaptation part. Despite this fact, the findings in the existing studies are advantageous for novel class detection modules. However, the issue of novel class arrival and class evolution can only be resolved using a complete framework which possesses the capability to: (1) detect novel classes and novel samples in existing classes (class evolution), (2) train the detected feature on-the-fly, (3) update the classifier without offloading and (4) be able to address this issue in high dimensionality feature space.

### Problem Formulation

The statistical properties of input data may vary at different time-steps (additional features can arrive), like when a new class appears in the stream (novel class arrival) or some new features appear in the existing classes (class evolution) which never have been seen (trained) by the classifier. Therefore, the classifier will misclassify the newly arrived samples or its performance will be degraded in samples with additional features. In [11], this issue has been discussed as a potential concept drift in imagery streams. However, the problem is that assuming the learner (M) at time t (Mt) trains with a given training data $D\;={\left\{\left(x\;i\;,y\;i\right)\right\}}_{t=1}^{m}$, where xi ∈ Rd is a sample instance x=(x1,x2,……, xm) and yi ∈ associated class labels y=(y1,y2,……, yc). Furthermore, the M is deployed for non-stationary stream environment for classification, where streaming data $S\;={\left\{\left(x\;t\;,y\;t\right)\right\}}_{t=1}^{\infty }$, such as xt ∈ Rd and yt ∈ $y{}^{\prime }=\left(y1,y2..,\;yc,\;yc+1,\ldots ..,yc{}^{\prime }\right)$: (c′> c). The goal is to detect and update the changes observed in the stream such that M(xt)→y′. Note that for arbitrary classes Cm and Cn ∈ ${}^{\prime }$ (Cm ≠Cn) if {xi, xj} ∈ Cm and {xk} ∈ Cn, it is possible that ${\left|\left|x\;i-x\;k\right|\right|}_{2}^{}$ < ${\left|\left|x\;i-x\;j\right|\right|}_{2}^{}$. Similarly, in the case of class evolution, when the sample instances x=(x1,x2,……, xm) inside existing classes are changed it make the learner (M) ineffective, for example, streaming data $S\;={\left\{\left(x\;t\;,y\;t\right)\right\}}_{t=1}^{\infty }$, where xt ∈ Rd and xt=(x1,x2.., xm+1): (c′> c) and yt ∈ y=(y1,y2,……, yc). Based on this problem formulation, this study poses some relevant research questions (discussed in Table 1) and investigates their research objectives, which are discussed in the relevant sections.

## 3. Ameliorate Adaptive Convolutional Neural Network (CNN) Ensemble Framework

**Figure 1:**
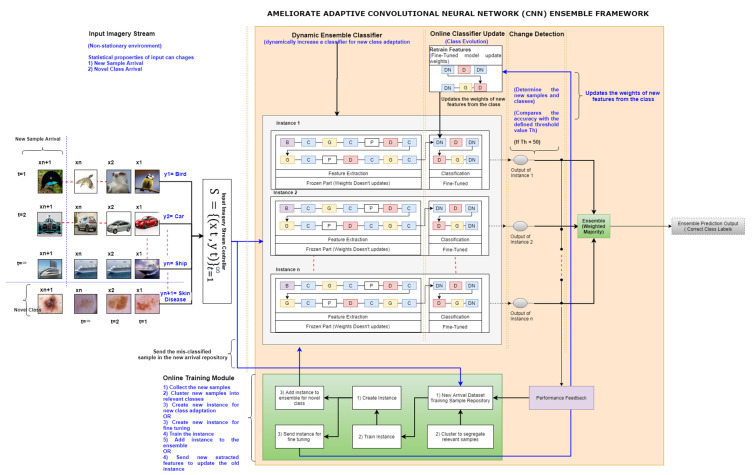
Ameliorated Adaptive Convolutional Neural Network (CNN) Ensemble framework to handle novel class and class evolution.

This section elaborates the proposed ameliorate adaptive CNN ensemble framework, as shown in Figure 1. The proposed ameliorated framework introduces significant improvements over previous adaptive CNN ensemble frameworks [13] (limited to new spectral band adaptation in online multispectral image classification) and handles the novel class and class evolution problems during online color image classification. In the ameliorated adaptive CNN ensemble framework, the authors used the diversity of the ensemble mechanism from a previous model (used to adapt new spectral bands), which helps handle the possible arrival of the new classes and samples.
**Algorithm 1:** Dynamic Ensemble Classifier Module of Ameliorated Adaptive CNN Ensemble Framework**Input:** The DEC module is an ensemble that possesses the instances I = (I1, I2,…..In). DEC is trained on training data contains classes such as, Cn_train_: (cn1, cn2,…….cni) and classifying the input sample from an imagery data stream DS. Such that DS possess input sample image S = (s1,s2…..sn) may belongs to classes Cn: (cn1, cn2,…….cni,,,,,,,,,cni+j,) at time t+1. Whereas samples from cni+j are unknown image sample to DEC module. **Initialization:** Th = 50 (threshold value for performance) 1: Counter:1 2: **While** data source > null /*/Valid input data source* 3: Classify (S), via single instance optimized CNN classifier [13] 4: Activate performance feedback module to determine the misclassified images 5: Determine the ensemble accuracies using voting 6: if % accuracy for S >= Th /*/if sample does not misclassify* 7: Repeat step 3, 4, 5 8: if % accuracy for S<=Th **//if sample misclassify** 9: Save the S /*/Save misclassified sample in training repository* *//as potential new classes or new samples in existing classes* 10: Counter++ 11: Repeat step 3, 4, 5 12: if counter = 200 *//number of misclassified instances reached to 200* 13: determine the possible new classes by using Algorithm 2. 14: Repeat step 3 15: **End while** **Output:** DEC module with (i_n+1)_ instances, and performing classification using Cni+j.

More precisely, the proposed novel ensemble approach contributes the diversity to the ensemble system in a simple yet effective manner. Also, the authors of this study have used the same single instance optimized CNN model [13] (carefully designed and fine-tuned during many experiments) as an instance of the ensemble. Further, they trained the instance using the CIFAR10 and ISIC 2019 (skin disease) datasets. The ameliorated adaptive CNN ensemble framework contains two core contributions: (1) an online training (OT) module and (2) an online classifier updater (OCU) module that majorly contribute to handle the novel class and class evolution problem. The authors of this study also have tweaked the internal structure of the dynamic ensemble classifier (DEC) of the previous framework, defined in Algorithm 1. The detailed steps of OT and OCU modules are presented in Algorithms 2 and 3, respectively.
**Algorithm 2:** Online Training Module of Ameliorate Adaptive CNN Ensemble FrameworkAssuming online image classification system has misclassified image samples, which are stored in the new sample repository x1, x2, x3,……xn belongs to classes y1, y2,…..yn, which are unknown. Each x contains the features f1, f2, f3…. fn. Also, the number of possible clusters K > 0. **Initialization:** 1: Determine the value of K, 2: Keep the centroids in the sample repository at random feature space 3: Let image samples clusters by k-means clustering into K cluster, where K > 0 4: Measure of well assigned the ith data point is to its cluster by below equation; $a\left(i\right)=\;\frac{1}{C\left(i\right)-1}\;\sum C\left(i\right),\;i\neq d\left(i,\;j\right)$ *//C(i): The clusters are assigned to the ith sample data point* *//|C(i)|: The number of sample data points in the cluster are marked to the ith data point* 5: Defined as the average dissimilarity to the closest cluster which is not it’s the cluster $b\left(i\right)=\min i\neq j(\frac{1}{C\left(j\right)}\;\sum j\;€C\left(j\right)\;d\left(i,\;j\right)$ 6: Determine the Silhouette coefficient s(i) is given by $s\left(i\right)\frac{b\left(i\right)-a\left(i\right)}{\max\left(a\left(i\right),\;b\left(i\right)\right)}$ *//Determine the average Silhouette for each value of k, and the value of k that has the maximum value of* *//s(i) is considered the optimal, number of clusters for the unsupervised learning algorithm.* 7: K <- Max(s(i)) /*/K=maximum value of s(i)* 8: Put K points randomly (as the initial centroids) from the image dataset 9: Calculate Euclidean distance of each point (image in the dataset) by below formula /*/with the identified K points (cluster centroids).* *//In general, for an n-dimensional space, the distance is* 10: Rearrange the image data point to the closest centroid /*/using the Euclidean distance* 11: Update the centroid position *//by taking the average of the image data points in each cluster group* 12: Repeat step 8, 9 and 10 till the centroids don’t upgrade 13: Segregate the obtained clusters image data into hypothetical classes, such as num. of clusters = number of classes i.e., Cluster n <- Yn(x) <- (x1, x2, x3,…..xi) /*/such as if three (3) clusters then three hypothetical classes will be created* 14: Create a new optimized CNN instance, where the number of instances = number of clusters 15: Train each new instances with each newly identified classes 16: Determine novel class or class evolution If Yn <- Yoldn the update Yold <- Yn using Algorithm 3. Else Add instance to the DEC module 17: End

**Algorithm 3:** Online Classifier Update Module of Ameliorate Adaptive CNN Ensemble FrameworkThis algorithm outlines the Online Classifier Update module for existing instance updating on the new sample. A new optimized CNN classification instance I_new_, Such as I_new_ is trained on new obtained classes. Such that streaming data $S\;={\left\{\left(x\;t\;,y\;t\right)\right\}}_{t=1}^{\infty }$, where xt ∈ Rd and xt=(x1,x2.., xm+1): (c′> c) and yt ∈ y=(y1,y2,……, yc). And Iold is old optimized CNN classification instance. Whereas C_n_ is the are new classes obtained posses the dataset Dc=(d1,d2.., dm Hence, Updated existing optimized CNN instance I_updated_, Such as I_updated_ <- I_old_ + I_new_ **Input:** A new optimized CNN classification instance I_new_ **Output:** Updated existing optimized CNN instance I_updated_ 1: Determine the I_old_ from the DEC module /*/select the appropriate old instance to be updated,* 2: Train I_new_ with C_n_, such as C_n_ contains Dc=(d1,d2.., dm) /*/train the new classification instance with the new obtained class* 3: Compare the weight of I_old_ and I_new_ *//using the weight difference algorithm* 4: Update the weight of I_old_ with I_new,_ such as I_updated_ <- I_old_ + I_new_ 5: End

### 3.1. Online Training Module

Fundamentally, the online training (OT) module segregates the misclassified image samples into the relevant classes. For that reason, this study has selected an unsupervised learning strategy to identify the possible classes from the available sample images. This study uses K-means clustering to determine the potential number of clusters (K) from the unknown data samples. However, to determine the initial value of K, the authors have of this study have applied the elbow and silhouette method and chosen the silhouette method due to its better performance. The silhouette is a measure of how close each point in one cluster is to points in the neighboring clusters and thus determine a more appropriate number of clusters. The coefficient varies between −1 and 1, which implies the nearest distance of that data point (image) and determine its actual cluster (1 implies that the instance is close to its cluster is a part of the right cluster, and −1 means that the value is assigned to the wrong cluster). The authors of this study have observed that silhouette is more accurate than elbow method (makes the decision regarding the optimal number of clusters more meaningful and clearer) but is computation expensive (as the coefficient is calculated for every instance). After determining the optimized value of K, they applied the K-means clustering algorithm and used the cosine and Euclidean distance method.

### 3.2. Online Classifier Update Module

The online classifier update (OCU) approach is inspired by the fine-tuning method of transfer learning. The run-time learning in the classifiers mostly done by dynamic neuron addition and weight updates. However, the proposed approaches are not validated on complex and high-dimensional data streams. In the OCU module, the authors of this study have updated the weights of the existing instance classifier (trained on old data) by replacing the classification part (layers which work on classification tasks).

**Figure 2:**
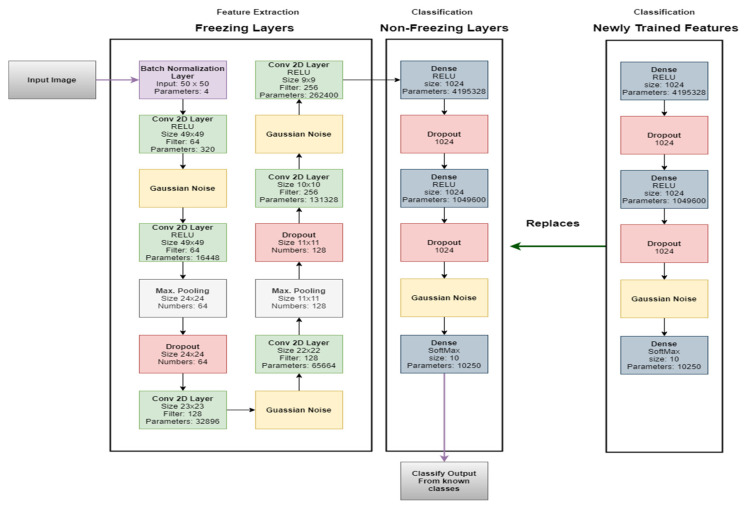
The process to update the existing classifier with new weights to handle class evolution.

The proposed approach is specially tuned for the high-dimensional data streams and works effectively for sophisticated features. The aim is to update the existing classification weight into newly obtained classification weight (obtained from Algorithm 1). The authors of this study have introduced a copy instance which possesses the new weight of the classification instance (trained on new classes). Also, they have used the weight difference techniques to determine that the newly trained instance is for updating the existing instance (classifier in the ensemble) or for adding new instance (classifier) in ensemble. The authors of this study found that for more complex classes the weight difference techniques did not work satisfactorily, and they used some manual intervention to inform the system either update the existing instance or add new instance in the ensemble. Algorithm 3 outlines the steps to follow to perform classifier update task, as shown in Figure 2.

**Figure 3:**
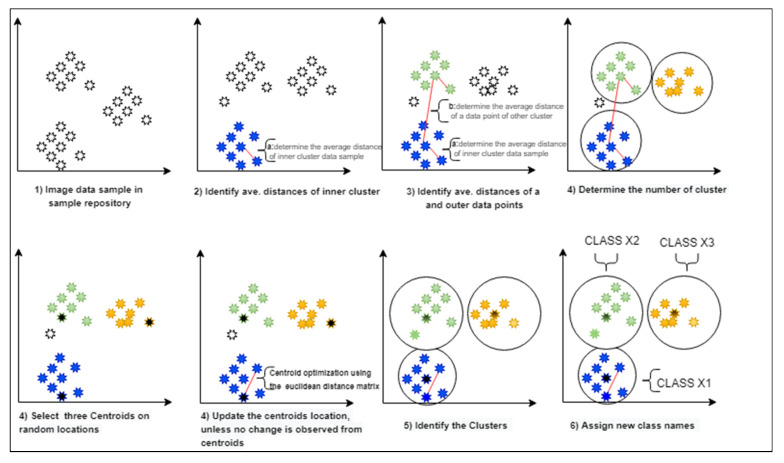
A proposed clustering approach to determine the new classes from the unknown image samples.

Therefore, the authors used some manual intervention for sophisticated features. Finally, from OCU module, the updated weights of the classification section are transferred to older instances (respective classifier in the ensemble), using Algorithm 3. Furthermore, they found that Euclidean distance method is more promising than the cosine distance matrix, hence Euclidean distance was selected as the primary distance measure during clustering. This OT module process segregates the misclassified image samples into the different relevant clusters. The obtained clusters are formed by the image similarity index by measuring, improving the centroids and comparing (the data points) using the Euclidean distance. Later, the segregated image samples for each cluster are separated with a given hypothetical class name, such as X1, X2,…, Xn, as depicted in Figure 3. Then OT module creates the new instances and train them for newly obtained classes (the number of new instances are equal to obtained new classes). Algorithm 2 defines the detail steps of proposed OT module.

## 4. Experimental Results

This section presents three (3) subsections to validate the effectiveness and performance of the proposed ameliorated framework, which are: (1) Section 4.1 details the data preparation and transformation of the datasets (2) Section 4.2 presents the experimental criteria and experimental setup; and (3) Section 4.3 and Section 4.4 displays the obtained results and performs the analysis and deduction.

### 4.1. Data Preparation and Transformation

To evaluate the performance of classification models, the common practice of researchers is to examine the proposed solution on benchmark and real datasets. Therefore, to validate the proposed framework, in this study, the authors have selected CIFAR10 and ISIC 2019: Skin disease (real dataset) as benchmark. CIFAR10 dataset is considered as one of the primary benchmark datasets for image classification task [25]. Furthermore, the primary intention to use ISIC 2019 is to verify the proposed framework with a real and challenging dataset (ISIC skin disease dataset is considered one of the most challenging due to its sophisticated features).

#### 4.1.1. CIFAR 10 Stream Pipeline Preparation to Simulate Concept Drift (CD)

**Figure 4:**
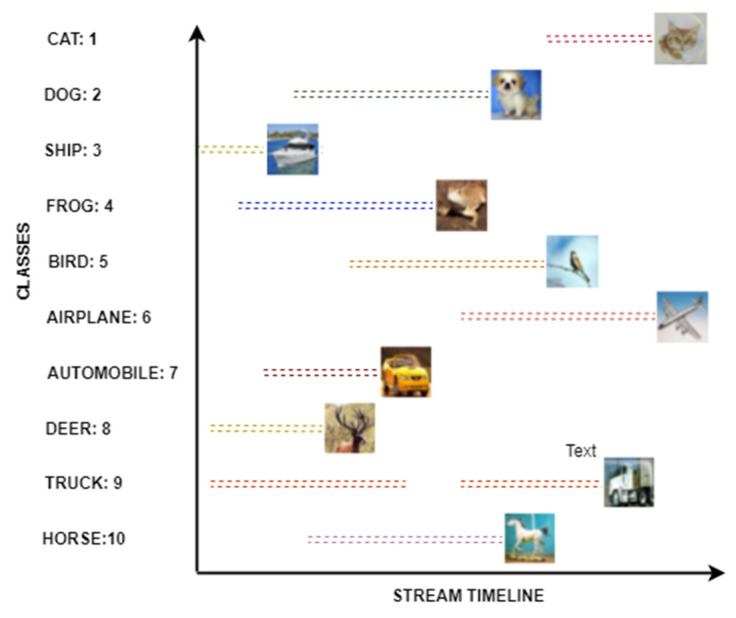
CIFAR10 dataset stream pipeline to simulate the concept drift scenarios.

The CIFAR10 dataset contains the real object images of ten (10) different classes, such as aeroplane, automobile, bird, cat, deer, dog, frog, horse, ship, and truck. Concretely, past studies have focused on two essential challenges when performing classification over a data stream which are; (1) concept drift [26] and (2) concept evolution [19,27]. Several studies have developed imagery stream pipelines using the CIFAR10 dataset to simulate the possible concept drift condition. Therefore, this study also adopted a similar strategy to demonstrate the novel class and class evolution issue, as shown in Figure 4.

#### 4.1.2. Skin Disease Data Stream Pipeline Preparation to Simulate Concept Drift (CD)

**Figure 5:**
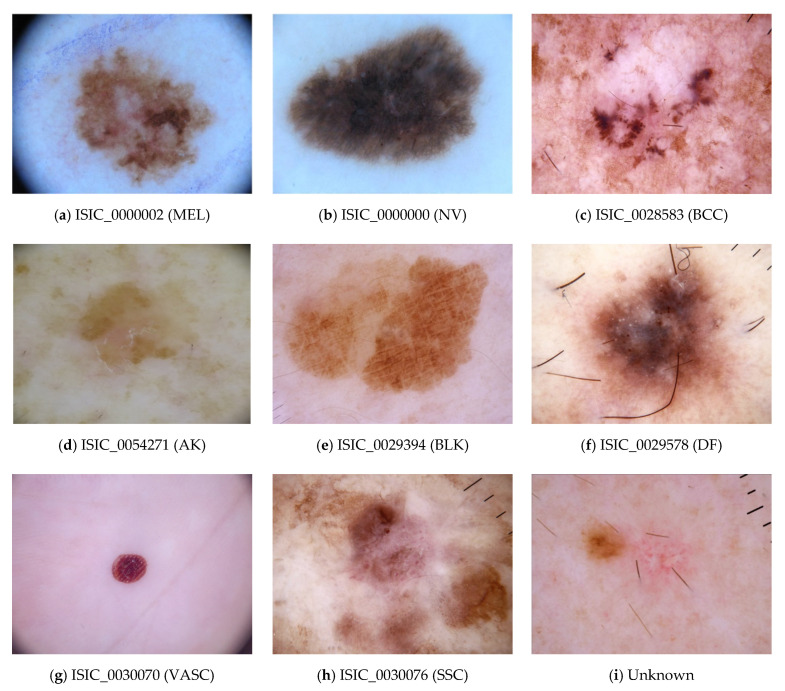
ISIC Skin Disease dataset. (**a**) Melanoma (MEL), (**b**) Melanocytic nevus class category (NV), (**c**) Basal cell carcinoma (BCC), (**d**) Actinic keratosis (AK) (**e**) Benign keratosis (BKL) (**f**) Dermatofibroma (DF) (**g**) Vascular lesion (VASC) (**h**) Squamous cell carcinoma (SSC) (**i**) Unknow Disease.

In this study, the authors of this study have selected a challenging real skin disease dataset created by the International Skin Imaging Collaboration (ISIC). ISIC released this dataset to the research and professional communities for open competition (Skin Lesion Analysis Towards Melanoma Detection) in 2019 (https://challenge2019.isic-archive.com/). The provided data is imbalanced across the classes and contains several similar features, which make this dataset more challenging for classification tasks. This dataset is an international repository of dermoscopic images for clinical testing and research toward automated algorithmic analysis. In total, the dataset contains the 25,331 dermoscopy samples divided into nine (9) classes, as shown in Figure 5.

**Figure 6:**
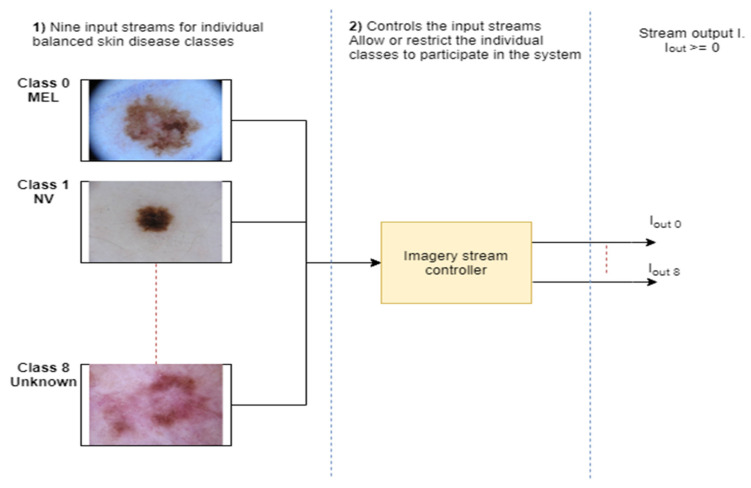
Skin disease dataset stream to simulate the concept drift scenarios.

The maximum number of samples is 12,875 (for class 1), and the minimum number of samples is 239 (for class 5), which represents the highly imbalanced classes. In addition, for each class the available samples are 4522, 12,875, 3323, 867, 2624, 239, 253, 628 in classes 0, 1, 2, 3, 4, 5, 6, 7, respectively. The class imbalance problem will cause overfitting issues (bias for the classes where the number of samples is more). Therefore, to handle the overfitting issue, the authors of this study have used an image augmentation technique which balances the number of classes in each class. The authors of this study have adopted the image augmentation technique to balance the imbalanced classes. Also, they have used the Python libraries with appropriate parameters to increase the image samples in each class and make them balance. Here, they also normalized the intensity values of the pixels of the image from 0 to 255 to 0 to 1 to reduce the computational complexity (normalization is important because feature scaling makes all features contribute equally during the gradient descent procedure, making optimization faster). Figure 6 presents the stream pipeline to simulate the concept drift scenarios. The stream pipeline has nine different streams (a stream for each class of skin disease) whereas the imagery stream controller module controls or manages the pipeline streams.

### 4.2. Experimental Criteria and Performance Measures

To simulate the novel class arrival and class evolution scenarios (where the new classes participate, or a class evolves in the systems), the authors of this study have separated the individual classes as individual streams, as shown in Figure 4 and Figure 6. Furthermore, it is necessary to report final accuracy on unseen input data. Therefore, they used cross-validation and holdout method and divided the dataset into training and testing with 3:1.

#### 4.2.1. Environment and Libraries

The experiments were carried out on the Google Cloud Platform (GCP) and Google Colaboratory. In the GCP server (us-west1-b region), authors of this study installed the Compute Engine Virtual Machine with additional machine learning and deep learning libraries. To speed up the complex computing jobs, authors of this used 16 vCPUs, 104 GB RAM with single NVIDIA GPU Tesla K80. The experiments implemented using the Python 3 programming language and the libraries listed below:

*Environment setup*:

(1) Python version (Python 3.6.3), installing from PyPI.
(2) The virtual environment from Anaconda
(3) TensorFlow (1.13), Theano and Keras (as backend) for complex deep learning classification.

*Libraries setup*:

(1) Scikit-learn library to perform basic machine learning tasks
(2) OpenCV to perform image processing tasks
(3) NumPy and Pandas for data manipulation and processing
(4) Seaborn and Matplotlib for visualization of the results

#### 4.2.2. Hyper-Parameter Optimization and Performance Measures

To select hyper-parameters for training a model, the authors of this study used a manual search strategy [28]. Through the manual search strategy, authors of this study acquired the optimized training hyper-parameters after various tuning iterations, as shown in Table 2. Besides, they also followed best practices referred by the research community—for example, selection of optimization function (Adam) and selection of cross-entropy (one-hot encoded). The classification accuracy is considered the most suitable metric to evaluate model performance in non-stationary environment [29]. In this study, they also have used performance measures (accuracy, loss, f1 score, precision, recall and ROC curve), which are recognized as primary classification performance indicators by the research community [30].

### 4.3. Experimental Results and Discussion

The details of the experimental results are discussed below. The authors of this study have performed the three experiments to analyze the performance of proposed framework. Firstly, experiment 1 evaluates the proposed framework performance for the novel class arrival issue. Secondly, experiment 2 verifies the effectiveness of the framework for class evaluation issue, experiment 3 compares the classification performance of the proposed framework with the state-of-the-art shallow learning and deep learning models, which are discussed below.

#### 4.3.1. Experiment 1: Performance in Novel Class Arrival

Experiment 1 formulates three different test cases (case 1, case 2 and case 3). A variety of test cases is essential to determine the effectiveness of the proposed framework under different stages during the novel class arrival. Such as, case 1 represents that the model is tested on the already trained dataset (no novel class arrival). Case 2 represents the arrival of new classes which were not trained previously on the models. In this case, the model did not apply the adaptation process. Case 3 shows, new classes arrived, which were not previously trained on the models. In this case, the model applied the adaptation process, as shown in Table 3. Moreover, two different models of the proposed framework instances were initiated, such as model1_CF10 for CIFAR10 data and model2_SD for ISIC skin disease data.

##### Case 1

Initially, the proposed framework is trained by the first five (5) classes of the CIFAR10 dataset (dog, frog, horse, ship truck) in offline/batch mode. The primary intension of this experiment was to evaluate the performance of the proposed framework under stable conditions. The obtained results were promising, with a recorded classification accuracy of 95.6%, loss 2.50, and 0.95 precision and recall. After arrival of novel class, the model (Model1_CF10) successfully adapt the new classes with classification accuracy 89% with the obtained loss 3.5, precision and recall 0.90 and 0.91 respectively, as shown in Table 4 and Table 5. Later, the authors of this study analyzed the performance of the proposed framework with a more challenging dataset (with the imbalance class samples and complex features) such as the ISIC skin disease dataset. Here, the proposed framework is trained by the first four classes of the skin disease dataset, which are dermatofibroma (DF), Vascular lesion (VASC), squamous cell carcinoma (SSC). Despite the complex features and class imbalance problem in skin disease dataset, the performance of the model is satisfactory, even better than highlighted in the literature. The obtained classification accuracy was 79% with the obtained loss, precision and recalled 0.40, 0.75 and 0.72, respectively. However, after arrival of novel class, the model (Model2_SD) successfully adapt the new classes with classification accuracy 70% with the obtained loss 0.92, precision and recall 0.67 and 0.64 respectively, as shown in Table 4 and Table 5.

**Figure 7:**
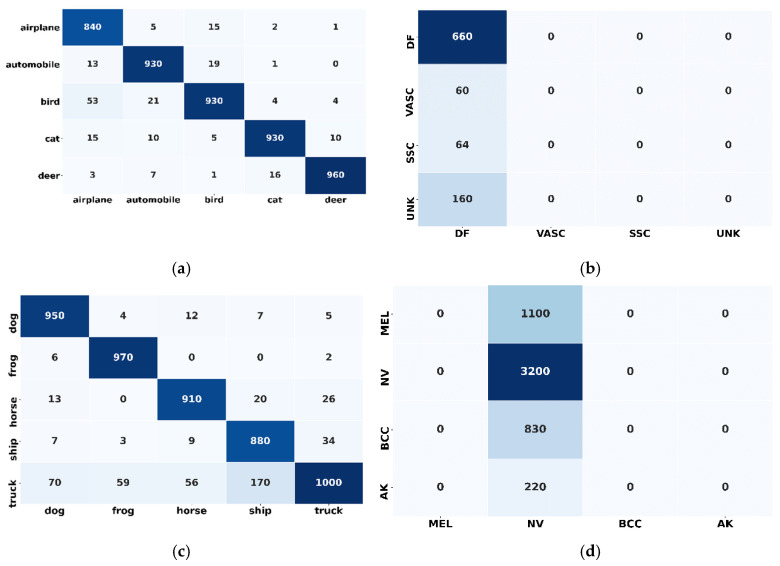
Confusion matrix: (**a**) Confusion matrix of model1_CF10 before novel class arrival; (**b**) Confusion matrix of model2_SD before novel class arrival; (**c**) Confusion matrix of model1_CF10 after novel class arrival; (**d**) Confusion matrix of model2_SD after novel class arrival.

Interestingly, the classification accuracy for the truck class (CIFAR 10) was reported as above 96%, and a 85% classification accuracy was found for the dog class (minimum accuracy). In contrast, the recorded classification accuracy of all other classes was above 93%, as shown in Figure 7a. Unlike the better performance of individual classes in the CIFAR 10 dataset, skin disease individual classes did not outperform and the maximum classification accuracy of 79% was reported for the dermatofibroma (DF) class. Further, all other classes performance is not satisfactory, as shown in Figure 7b.

##### Case 2

In case 2, to the proposed framework (model1_CF10) (which was trained on the first five classes, correctly classifying the samples) five new classes have been introduced (which were not trained on the proposed framework). These five (5) classes (aeroplane, automobile, bird, cat deer) cause a performance degradation in the proposed (ameliorated) framework. In this case, the adaptive feature of the proposed framework was deliberately kept off. It was essential to measure the performance degradation in the proposed framework after observing the novel classes. The results have validated the problem formulation, with a noticeable decrease in classification accuracy such as 63% (which was 95.6% in case 1). Similar is the case with precision and recall. A 30% to 40% decrease in precision and recall is observed, such as 0.60 (0.95 reported in case 1) and 0.50 precision (0.95 reported in case 1). Similarly, to measure the performance degradation in the proposed framework (model2_SD), the authors of this study have introduced four (4) new classes in the input stream, such as melanoma (MEL), melanocytic nevus class category (NV), basal cell carcinoma (BCC), and actinic keratosis (AK). In this scenario, the deployed framework found 59% classification accuracy after observing four (4) new classes. Its classification accuracy decreased by up to 20% (reported 79% in case 1). Similar is the case with loss, precision and recall, as shown in Table 4 and Table 5.

##### Case 3

In case 3, five new classes (not trained on the proposed framework) have been introduced to the proposed framework (model1_CF10) (which was trained on the first five classes, and correctly classified the samples). These five (5) classes (aeroplane, automobile, bird, cat deer) caused a performance degradation in the deployed proposed framework. Contrary to case 2, in this case, the adaptive feature (the ability to adapt the novel class on-the-fly) of the proposed framework was on. The intuition behind this experiment was to determine the adaptability feature effectiveness of the proposed framework. It can be shown that the proposed framework outperformed and successfully achieved a gain in classification accuracy of more than 25%. After adapting the new classes, the reported classification accuracy is 89% (63.5% in case 2). Also, an improvement in precision and recall is noticed. However, the loss, in this case, is increased to 3.5, which was 2.5 and 1.29 in case 1 and case 2, respectively, since a model training in offline mode will always be better than in the online mode. In the case of model2_SD, the proposed framework performance did not increase substantially. Through the obtained results, it can be noticed that only an 11% improvement in the model2_SD has been recorded after novel class adaptation, although the results are also promising and show that the proposed framework is working well (not outperforming) with complex features and under class imbalance situation. Further, unlike model2_SD, the performance of individual classes in model1_CF10 is found to be better, such as for the classes deer and automobile for which the reported individual classification accuracies are 97% and 98%, respectively, which is even higher than the composite classification accuracy (89%). Besides, all the individual classification accuracies are reported above 90% except the cat class, which has 89% accuracy, as shown in Figure 7c. The individual classes in model1_SD does not outperform, and the performance on other classes is not satisfactory, as shown in Figure 7d.

**Figure 8:**
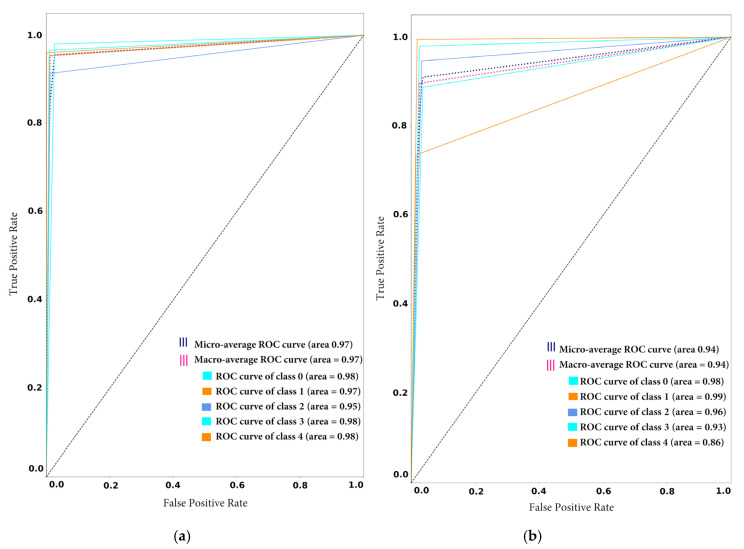

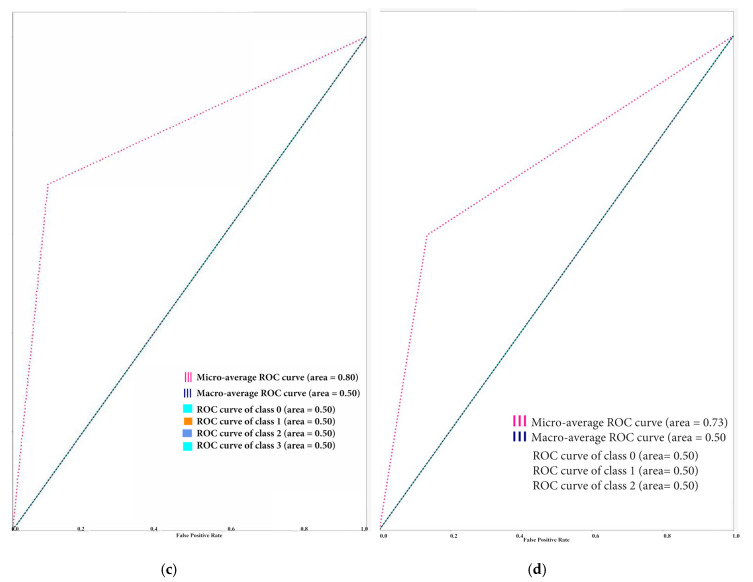
Receiver Operating Characteristic (ROC) curve: (**a**) ROC curve of model1_CF10 before novel class arrival (**b**) ROC curve of model1_CF10 after novel class arrival (**c**) ROC curve of model2_SD before novel class arrival (**d**) ROC curve of model2_SD after novel class arrival.

To demonstrate the tradeoff between sensitivity and specificity, the authors of this study also drew receiver operating characteristic (ROC) curve plots to see the possible increase in sensitivity accompanied by a decrease in specificity in the proposed framework before and after novel class arrival. As depicted in Figure 8a,b, the ROC curve is closer to the left-hand border and then the top border of the ROC space, which shows the true positive rate against the false-positive rate. In the case of model1_CF10, the obtained ROC curve is desirable, as shown in Figure 8a,b. However, in the case of model2_SD, the ROC curve comes to the 45-degree diagonal of the ROC space, which demonstrates less accuracy of the test, as shown in Figure 8c,d.

#### 4.3.2. Experiment 2: Validate the Online Fine-Tuning Performance of the Proposed Framework When New Samples are Observed

**Figure 9:**
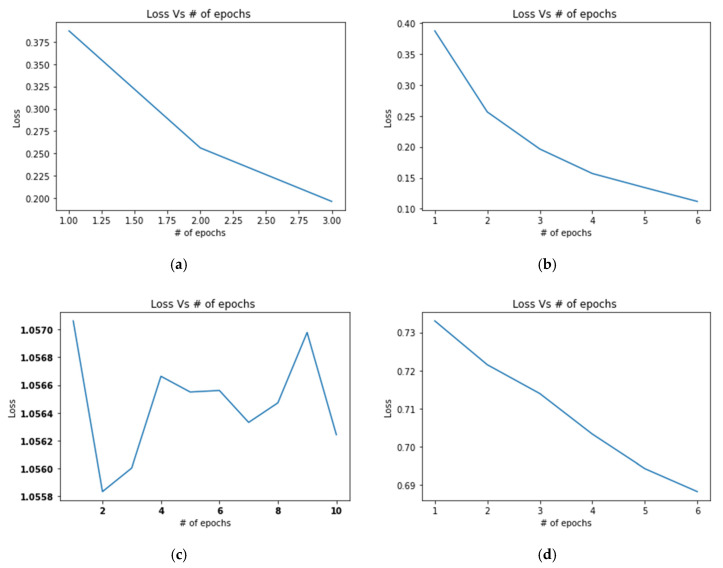
Training loss and number of EPOCs: (**a**) Before new sample arrival for model1_CF10; (**b**) After new sample arrival for model1_CF10; (**c**) Before new sample arrival for model2_SD; (**d**) After new sample arrival for model2_SD.

In Experiment 2, the authors of this study have trained the model1_CF10 and model2_SD with partial image samples of all the available classes of CIFAR10 and ISIC 2019 (Skin disease), respectively. However, during initial training (offline) from each class 40% of image samples with some unique characteristics were segregated separately and the models are trained on the other 60% of the sample data available in each class. After model deployment (model1_CF10 and model2_SD), the stream contains 100% of the dataset from the CIFAR10 and ISIC skin disease data stream. Here, the model detected the newly arrived samples, determined the new feature spaces in the existing classes and fine-tuned the existing classifier with newly arrived batch samples. Figure 9a, presents the correlation of reported loss and EPOCs during offline training, and Figure 9b presents the correlation of reported loss and EPOCs after online fine-tuning (using the online classifier update (OCU) module). It can be shown that in online fine-tuning, a continuous decrease in loss is observed (which is desirable). After six (6) EPOCs, the obtained loss for model1_CF10 is less than 0.11 approximately, which is better than in offline learning. Interestingly, for the model2_SD, the offline training loss showed several fluctuations after each EPOC, and the minimum loss in above 1, as shown in Figure 9c. Furthermore, it can be noticed that the reported loss is not stable (also tried to train the model on higher EPOCs). On the contrary, the online fine-tuning works well for model2_SD (better than offline) and its loss continuously decreases after each EPOC. For example, the final loss is 0.65 approximately (minimum loss reported in offline was 1), which is much better than offline learning) as shown in Figure 9d.

#### 4.3.3. Experiment 3: Comparative Analysis of Proposed Framework with State-of-the-Art Shallow Learning and Deep Learning Models

This experiment compares the performance of the proposed framework with the state-of-art shallow learning and deep learning models under the novel class arrival issue. The authors of this study have compared proposed framework with ensemble convolutional neural network (CNN), ensemble support vector machine (SVM) and random forest models. These models are widely used for concept drift handling [31]. In this experiment, two experimental scenarios were simulated: (1) case 1, when the model is testing with no novel class arrival issue and, (2) case 2 when five (5) new classes participated in the stream to be classified. It can be seen from Table 6 that under stable conditions, the performance of all the models was satisfactory and above 95% approximately. However, the random forest classification accuracy was the highest among all models (which is 96.84%). On the contrary, when the novel classes participated in the imagery streams (to be classified) the all state-of-art models badly performed and their classification accuracy was drastically degraded by as much as 50% approximately, whereas, the proposed framework outperformed under the novel class arrival conditions. The dynamic and adaptability feature in proposed framework avoided the massive performance degradation in the model, and its classification accuracy only decreased by 6.6%. Such as, after adaptation, the proposed framework maintained its classification accuracy up to 89% with the obtained loss 3.50, and f1-score 0.90, as shown in Table 6. Interestingly, the other performance parameters of proposed framework, such as loss and F1-score, are also found much satisfactory as compared to other state-of-the-art models, as shown in Table 6.

##### Comparative Analysis before the Arrival of Novel Classes (Confusion Matrix and ROC Curve)

**Figure 10:**
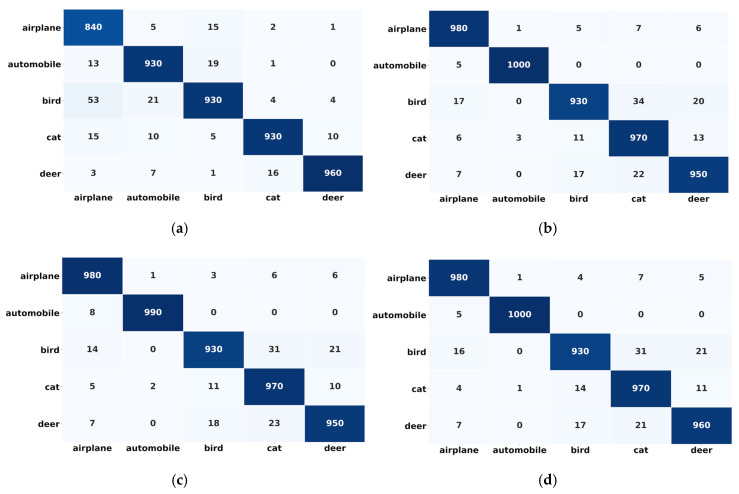
Confusion matrix of proposed framework and state-of-the-art shallow learning and deep learning approaches after novel class arrival issue: (**a**) Confusion matrix for ameliorated adaptive CNN ensemble (proposed framework); (**b**) Confusion matrix for Ensemble CNN; (**c**) Confusion matrix for Ensemble Support Vector Machine; (**d**) Confusion matrix for Random Forest.

**Figure 11:**
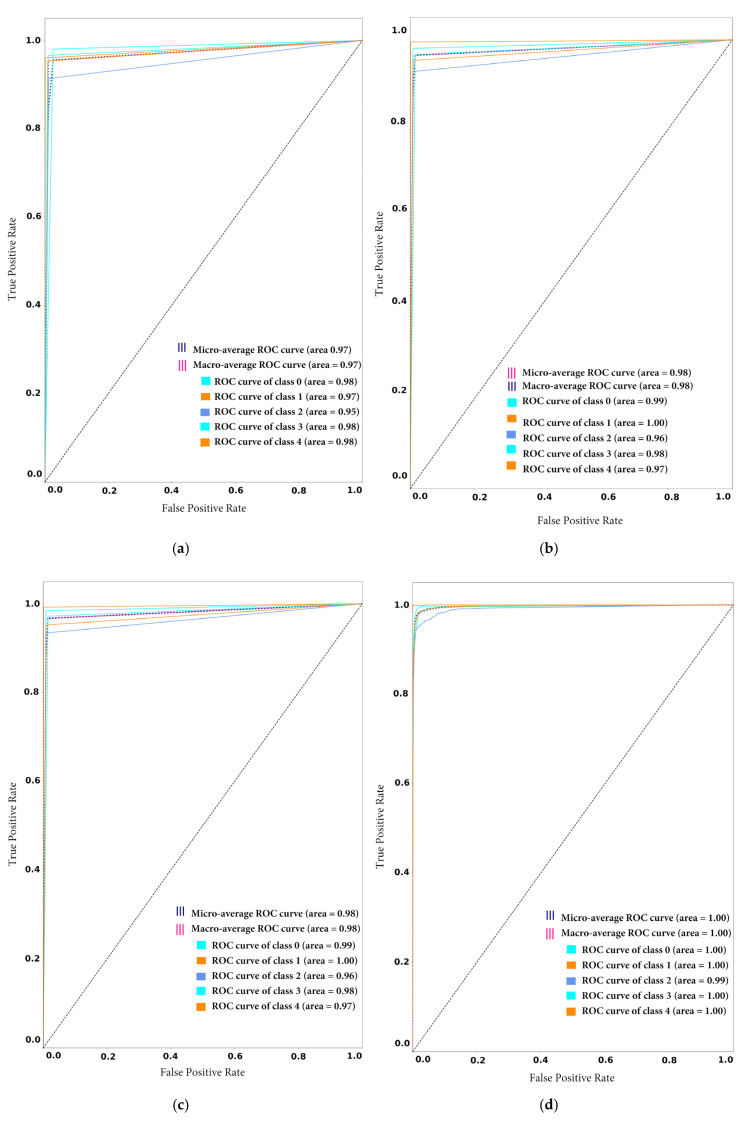
Receiver Operating Characteristic (ROC) curve on stable condition: (**a**) ROC curve of proposed framework (**b**) ROC curve of Ensemble CNN (**c**) ROC curve of Ensemble SVM (**d**) ROC curve of Random Forest.

The confusion matrix analysis and ROC curve give more details about the performance of the model. Figure 10 and Figure 11 show the classification performance of the individual participating classes.

From Figure 10a–d it can be noticed that all state-of-the-art models outperformed for the individual classes and their classification accuracy reached above 90%. However, in the case of proposed framework, one class (truck) achieved around 85% of accuracy.

Also Figure 11a–d show that the random forest model classification is best among all the models with a micro and macro average ROC value equal to 1. Also, the all ROC curves of all individual classes (0,1,2,3 and 4) are higher than for all other models. For example, Figure 11d depicts the ROC value of class 0,1,3,4 as 1 (which is maximum) and the class 2 ROC value is 0.98. On the contrary, the proposed framework performance is the lowest among all models. Thus, from Figure 11a it can be observed that the micro and macro average ROC value is equal to 0.97, and all individual classes are higher than all other models. For example, Figure 11d depicts the ROC value of class 0, 1, 2, 3 and is between the range of 0.90 to 0.98 (which is minimum as compared to other participating models).

##### Comparative Analysis after the Arrival of Novel Classes (Confusion Matrix and ROC Curve)

**Figure 12:**
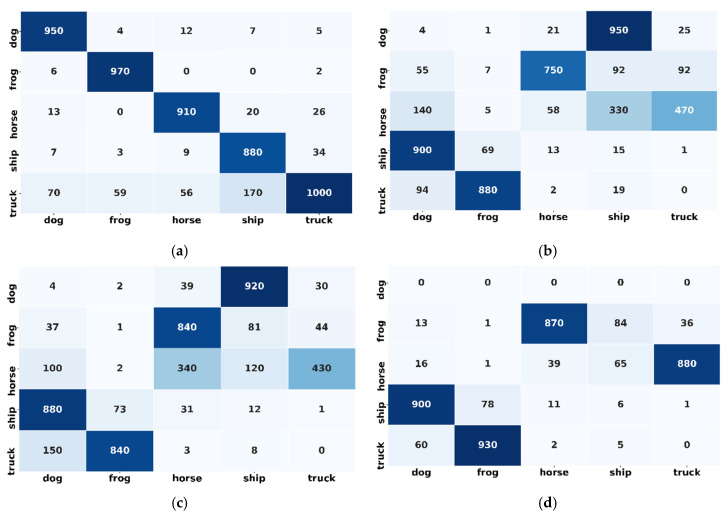
Confusion matrix of proposed framework and state-of-the-art shallow learning and deep learning approaches after novel class arrival issue: (**a**) Confusion matrix for ameliorated adaptive CNN ensemble (proposed framework); (**b**) Ensemble CNN; (**c**) Ensemble Support Vector Machine; (**d**) Random Forest.

**Figure 13:**
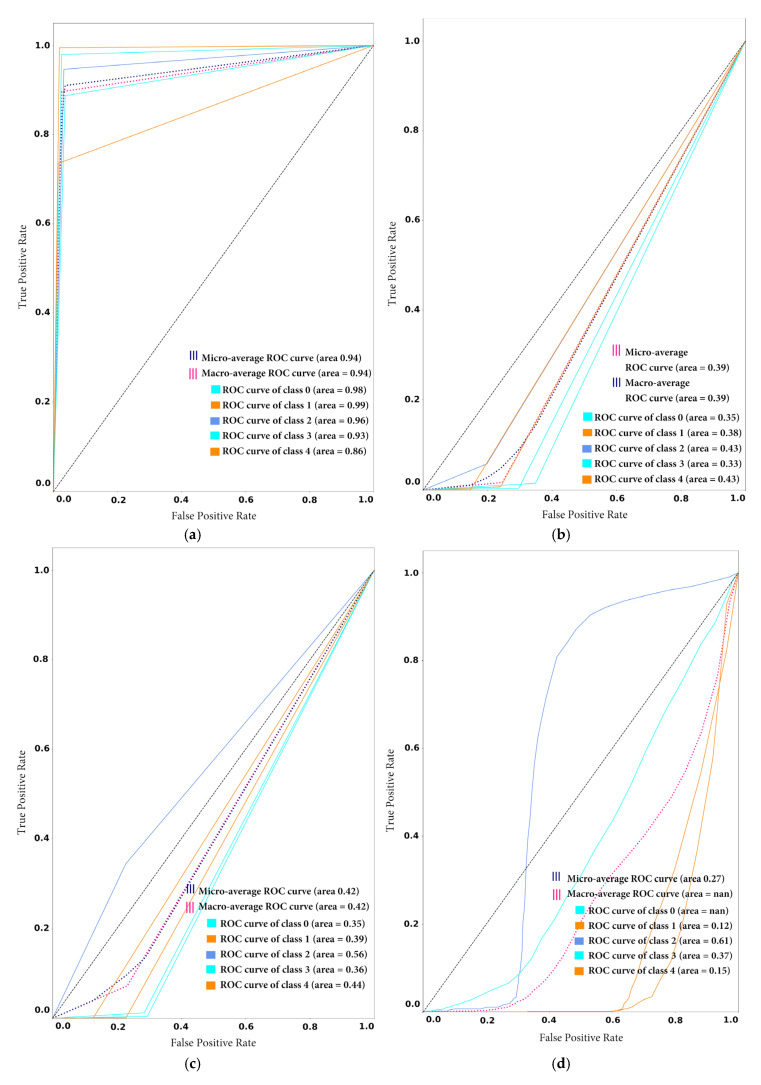
Receiver Operating Characteristic (ROC) curve after arrival new class: (**a**) ROC curve of proposed framework (**b**) ROC curve of Ensemble CNN (**c**) ROC curve of Ensemble SVM (**d**) ROC curve of Random Forest.

Figure 12 presents the confusion matrix for the participant’s models after the novel class arrival. Figure 12 depicts the classification performance of the individual participating classes; the results validate the effectiveness of proposed framework. For example, Figure 12a highlights the diagonal areas of the proposed framework confusion matrix, which confirms that the model outperformed for the individual classes. However, the classification performance of all other participating models is severely degraded for individual classes. The scattered values in the confusion matrix demonstrate the lousy performance of the ensemble CNN, ensemble SVM and random forest in Figure 12b–d, respectively. Also, Figure 13a shows that the proposed framework is the best among all the models with a micro and macro average ROC value is equal to 0.94. The ROC curve of all individual classes (0,1,2,3 and 4) is higher than all other models and are in the range of 0.85 to 0.97. Conversely, all other models of classification performance are worse. From Figure 13a–d it can be observed that the micro and macro average ROC value of all the other models are equal to 0.44, 0.42 and 0.35 for ensemble CNN, ensemble SVM and random forest, respectively.

### 4.4. Results Analysis and Deduction

*Experiment 1***:** This experiment demonstrates the performance of the proposed framework under novel class arrival conditions. The results showed a significant performance improvement after the novel class adaptation for the benchmark datastream (CIFAR10) and real datastream (ISIC 2019). The proposed framework increased the classification accuracy by more than 20% due to its effective adaptation solution. Both the model1_CF10 and model2_SD outperformed and avoid the classification performance degradation (in terms of accuracy) after observing and adapting novel classes. However, the authors of this study have noticed that the model2_SD is not markable in stable condition due to the sophisticated and similar features available in the ISIC 2019 (skin disease) dataset.

*Experiment 2:* This experiment validates the performance of the proposed online classifier update module (of the proposed framework) to handle the class evolution problem. The results have shown the effectiveness of the proposed approach for new sample adaptation on-the-fly. It can be observed the performance of both models (model1_CF10 and model2_SD) continuously improved with the decrease in learning loss. Furthermore, the model2_SD found several loss fluctuations during offline learning. On the contrary, in online learning mode, the model2_SD reduction of loss is smooth and significant after each new EPOC.

*Experiment 3:* The intuition behind Experiment 3 is to compare the performance of the proposed framework with state-of-the-art deep learning and shallow learning models. The acquired results provide some exciting findings. The performance of all the participated models is impressive under stable conditions (when no novel class arrives) and under stable conditions, the classification accuracy of the proposed framework is less than all other state-of-art models, especially random forest which performance is the best among all. On the contrary, only the proposed framework showed a substantial increase in performance (up to 89%) after novel class arrival and all the state-of-art models’ classification accuracy is reported as 50–55% approximately.

## 5. Conclusions and Future Work

This study addressed two (2) non-stationary data assumptions and temporal inconsistencies, (such as novel class and class evolution) for dynamic image classification. Also, this study proposed an ameliorated adaptive convolutional neural network (CNN) ensemble framework as an attempt to provide a generic approach for handling several kinds of temporal inconsistencies found in high-dimensionality data streams.

Previously an adaptive CNN ensemble framework was proposed for new spectral band adaptation during complex multispectral image classification. However, in this study, the authors have presented an improved version of previously proposed framework with the additional online training (OT) module and online classifier update (OCU) module to address the novel class arrival and class evolution issue (new sample arrival in the existing classes). An OT module is a clustering-based approach which uses the Euclidean distance and silhouette approach to determine the possible newly arrived classes, whereas, the OCU updates the current weights of the ensemble classifiers with newly arrived samples in existing classes. The proposed novel ensemble approach ensures the ensemble diversity and adaptability in a simple yet effective manner. Specifically, in the proposed ensemble approach, the single optimized CNN classifier (instance) handles the novel class arrival issue.

The proposed framework showed satisfactory performance under non-stationary scenarios using the benchmark and real data streams and outperformed against state-of-art models. The results have demonstrated the significant improvement (more than 20%) in proposed framework classification accuracy after the novel class adaptation and class evolution issue were resolved. The authors of this study also have tested proposed model (framework) using ISIC 2019 challenge skin disease dataset. The authors of this study have noticed that the performance of the proposed framework is not remarkable under stable conditions but much better than the performance of a state-of-the-art model. Thus 70% to 79% classification accuracy was observed due to the sophisticated and similar features available in the ISIC dataset. In future work, the authors aim to develop an Internet of Things (IoT)-enabled adaptive intelligent dermoscopy device (for dermatologists) which demands more accurate classification accuracy (95–97%). Hence the improvement in the classification accuracy for the similar and complex feature-based classes is the future concern of this study.

## Figures and Tables

**Table 1:** The raised research questions (RQ’s), research objectives (RO’s) and relevant section.

Research Questions	Research Objectives	Section
**RQ1:** How to handle the issue of novel class arrival and class evolution in the non-stationary high-dimensional stream?	**RO1:** To propose an ameliorate (improved) version of adapted CNN ensemble (online training and online classifier update) using optimized clustering approach to handle novel class and class evolution issue in online imagery stream.	Section 3
**RQ2:** How to ensure the classification performance of the model after noticing a novel class and class evolution?	**RO2:** To validate the classification performance of the proposed framework after novel class and class evolution using the benchmark and real dataset (a challenging dataset). **RO3:** To evaluate the performance of the proposed framework with state-of-art shallow learning (Ensemble SVM and Random Forest) and deep learning (Ensemble CNN).	Section 4

**Table 2:** Training hyper-parameters (Tuning values and optimized values).

Training Hyper-Parameters	Tuning Values	Optimized Values
Mini-Batch Size	16, 32, 64, 128, 256	120
Learning Rate	0.1, 0.01, 0.001	0.001
L1 regularization (Lambda Parameter)	0.001, 0.0003	0.0003
Number of EPOC	10–100	100
Optimization Function	Adam	Adam
Cross-Entropy	One-hot encoded	One-hot encoded

**Table 3:** Cases, scenarios and their description.

Cases	Scenarios	Description
**Case 1**	No novel class arrival	When the model is tested on the already trained dataset (No novel class arrival). In the case of CIFAR10, Classes 0–4 participated In the case of Skin Disease, Classes 0–3 participated
**Case 2**	Novel class arrival but before adaptation	When new classes (which were not already trained on the models) are arrived. In this case, the model did not apply the adaptation process. In the case of CIFAR10, Classes 5–9 participated In the case of Skin Disease Class, 4–7 participated
**Case 3**	Novel class arrival but before adaptation	When new classes (which were not already trained on the models) are arrived. In this case, the model applied the adaptation process. In the case of CIFAR10, Classes 5–9 participated In the case of Skin Disease Class, 4–7 participated

**Table 4:** Accuracy and loss for the CIFAR10 and ISIC 2019 (Skin disease) streams under different cases.

Model Configuration	Classification Accuracy (%)			Loss		
	Case 1	Case 2	Case 3	Case 1	Case 2	Case 3
**Model1_CF10**	95.60	63.50	89.00	2.50	1.29	**3.50**
**Model2_SD**	79.00	59.00	70.00	0.40	13.90	**0.92**

**Table 5:** Precision and recall for the CIFAR10 and ISIC skin disease data streams under different cases.

Model Configuration	Precision			Recall		
	Case 1	Case 2	Case 3	Case 1	Case 2	Case 3
**Model1_CF10**	0.95	0.50	0.90	0.95	0.60	**0.91**
**Model2_SD**	0.75	0.14	0.67	0.72	0.25	**0.64**

**Table 6:** Comparative analysis of the proposed framework and state-of-the-art models.

	Accuracy (%)		Loss		F1-Score	
Models	Case 1	Case 2	Case 1	Case 2	Case 1	Case 2
Proposed Framework	95.60	**89.00**	2.50	**3.50**	0.95	**0.90**
Ensemble CNN	96.00	50.96	1.17	7.0	0.96	0.49
Ensemble SVM	96.68	50.72	1.14	5.34	0..96	0.52
Random Forest	96.84	43.13	0.20	5.9	0.97	0.55

## Data Availability

The CIFAR 10 dataset is available at “https://www.cs.toronto.edu/~kriz/cifar.html” and the ISIC 2019 challenge dataset is available at “https://challenge2019.isic-archive.com/”.
